# Prospective and external evaluation of an AI model for continuous and early prediction of moderate and severe AKI in critically ill patients

**DOI:** 10.1186/s40635-026-00928-y

**Published:** 2026-06-10

**Authors:** Francesca Alfieri, Simone Zappalà, Alessandro Bacci, Valentina Cauda, Marco Basso, Gaia Musso, Luca Cochelli, Carmine Domenico Votta, Laura Mariconti, Laura Maderna, Gianluca Russo, Josep Gomez, Federico Esteban-Reboll, Mari Carmen Gilavert Cuevas, Maria Bodì, Stefano Finazzi, Kianoush Kashani, Andrea Ancona

**Affiliations:** 1 U-Care Medical SRL, Corso Castelfidardo 30A, 10129 Turin, Italy; 2 Department of Applied Science and Technology, Politecnico di Torino, C. So Duca degli Abruzzi 24, 10129 Turin, Italy; 3 Ospedale Maria Vittoria, Torino, Via Luigi Cibrario 72, Turin, Italy; 4 Department of Anaesthesia and Intensive Care, Ospedale Maggiore di Lodi - ASST Lodi, Via Donatori del Sangue, 1, 26900 Lodi, Italy; 5 Servicio de Medicina Intensiva, Hospital Universitario de Tarragona Joan XXIII, Carrer Dr. Mallafré Guasch, 4, 43005 Tarragona, Spain; 6 Universitat Rovira i Virgili, Av. Catalunya, 35, 43002 Tarragona, Spain; 7 Dipartimento di Salute Pubblica, Laboratorio di Clinical Data Science, Istituto di Ricerche Farmacologiche Mario Negri IRCCS, Ranica, Bergamo, Italy; 8 Division of Nephrology and Hypertension, Division of Pulmonary and Critical Care Medicine, Department of Medicine, Mayo Clinic, Rochester, MN USA

**Keywords:** Artificial intelligence, Acute kidney injury, Digital biomarker, Intensive care unit, Prospective validation

## Abstract

**Background:**

Acute kidney injury (AKI) is a major complication in critically ill patients, burdening both patients and healthcare systems. We previously introduced an AI-based model for early and continuous prediction of ICU-acquired AKI (ICU-A-AKI-2/3). In this study, we enhanced the model to better handle missing data, a common challenge in clinical settings. The upgraded model was validated in both retrospective and prospective cohorts, demonstrating improved robustness and predictive performance.

**Methods:**

The model was validated in retrospective cohorts from three countries (US, Netherlands, Italy; N = 70,107 ICU admissions) across 176 ICUs. It was then prospectively validated in three European hospitals (Italy, Spain; N = 329) from May to October 2023. Using an XGBoost classifier, the model analyzes clinical data from ICU patients to predict hourly risk probabilities for AKI stages 2 and 3, as defined by KDIGO.

**Results:**

In retrospective cohorts, the AI model achieved an auROC greater than 0.89 for early detection of ICU-A-AKI-2/3. Prospective validation showed auROCs between 0.82 (95% CI 0.73–0.92) and 0.96 (95% CI 0.92–0.99) across hospitals, with a mean lead time of approximately 14 h.

**Conclusions:**

This enhanced AI model offers timely prediction of ICU-A-AKI-2/3 episodes, as demonstrated across diverse cohorts. Its high predictive performance represents a significant advancement in integrating AI into clinical workflows, enhancing AKI management and improving clinical outcomes in ICU settings.

**Supplementary Information:**

The online version contains supplementary material available at 10.1186/s40635-026-00928-y.

## Introduction

Critically ill patients are susceptible to developing organ dysfunction during intensive care unit (ICU) admission. Acute kidney injury (AKI), a sudden and silent decrease in renal function, is a major complication linked to increased morbidity, mortality, and healthcare costs [1, 2]. Approximately one-fifth of hospitalized patients experience ICU-acquired AKI stage 2/3 KDIGO (ICU-A-AKI-2/3), 40% of whom are oliguric and face worse outcomes [3, 4]. From an economic perspective, managing AKI incurs high healthcare costs [5, 6].

Current AKI diagnostic criteria rely on urine output and serum creatinine changes, which fail to respond in a timely manner, leading to delayed recognition and suboptimal management. Late diagnosis of AKI and delayed management may be linked to an increased risk of poor renal recovery, resulting in significant short- and long-term consequences, such as a greater likelihood of progressing to chronic kidney disease and ultimately requiring dialysis or transplantation [7, 8].

Timely identification of patients with an increased risk of AKI can potentially enable proactive management approaches to mitigate AKI progression and its complications, improving clinical and operational outcomes in the ICU. In the past decade, with the advent of artificial intelligence (AI) and machine learning (ML) tools, the ability to predict clinical syndromes has significantly improved [9]. While several studies have focused on early AKI detection, many lack extensive prospective validation or real-world data or are affected by methodological biases such as retrospective design, single-center settings, or selection of highly specific patient populations, which limit the generalizability of their findings [10].

A key limitation of existing models is their inability to handle missing data, reducing their real-world applicability where some clinical parameters could be missing.

In this context, “early” and “timely” prediction refer to model alerts occurring sufficiently in advance of AKI onset to allow clinical intervention, as quantified by lead time. Although no universally accepted threshold exists, predictions occurring several hours before onset (up to approximately 24 h) are generally considered clinically actionable in ICU settings. We previously conducted studies with the aim of developing an AI-based model to predict the risk of ICU-A-AKI-2/3 during ICU admission. Deep and machine learning techniques were developed [11, 12] for oliguric and nonoliguric AKI, leveraging routinely collected clinical parameters, including laboratory values, demographic information, and vital signs [13].

Here, we present an enhanced model designed to address a key limitation of existing AKI prediction model: the management of missing clinical data. In this work, we transitioned from the previously used Random Forest to XGBoost, due to its native handling of missing values and strong performance on structured clinical data. The model was developed, tested, and externally validated in a large retrospective multicenter cohort of ICU patients. It was then integrated into the electronic health records systems of three ICUs for prospective validation in real-world scenarios.

## Materials and methods

### Data sources and inclusion criteria

The retrospective study cohorts were derived from four different sources in multiple centers in various countries. The databases included the Mimic-III database [14], which stores data from Beth Israel Deaconess Medical Center in Boston (53,423 admissions, 38,597 distinct patients from 2001 to 2012); the eICU collaborative research multicenter database [15], which contains data from more than 200 different United States ICUs (200,859 admissions, 139,367 patients from 2014–2015); the Amsterdam UMC database [16], which contains anonymized data from 20,109 European patients admitted to the university hospital ICU of Amsterdam in the Netherlands (23,106 admissions from 2003–2016); and the Margherita Tre database, developed by the Group for the Evaluation of Interventions in Intensive Care Medicine (GiViTI) [17, 18], which stores data from 27 different Italian centers (60,430 ICU admissions, 55,702 patients from 2001–2022). Prospective cohorts were recruited from Maggiore Hospital (Italy, Lodi), Hospital Universitario de Tarragona Joan XXIII (Spain, Tarragona) and Maria Vittoria Hospital (Italy, Turin). All prospective validation sites were general intensive care units, without a specific clinical specialization.

The use of Mimic-III was motivated by the need to ensure methodological continuity with the previously developed version of the model, allowing direct assessment of improvements without introducing confounding factors related to dataset differences.The inclusion criteria for the cohorts wereadults aged 18 years or older admitted to the participating ICUs. We excluded critically ill patients who were already receiving renal replacement therapy (RRT) at ICU admission or who had community-acquired AKI (CA-AKI), defined as stage 2 or 3 AKI at ICU admission.

Additionally, patients were excluded if they had missing data on weight, height, and age. For the retrospective cohort, patients were excluded if their urine output or serum creatinine data were missing in the first 12 h after ICU admission. For the prospective cohort, patients were excluded if these data were missing in the first 24 h after ICU admission, to increase patient enrollment and better reflect real-world data availability (where the first measurement of Serum Creatinine or Urine Output can happen also between the 12th and 24th hour after ICU admission). These exclusions were applied prior to analysis to ensure reliable AKI definition. The number of excluded patients in the prospective cohort is reported in Supplementary Material Suppl. Info (Section J, Table S13), and their exclusion may represent a potential source of selection bias. To evaluate the potential impact of this criteria, we performed a sensitivity analysis applying a 12-h window, consistent in the prospective cohort with the retrospective cohort.

Finally, patients with an ICU length of stay shorter than 24 h were excluded.

The study was conducted in accordance with the Declaration of Helsinki. Ethical approval was obtained from the relevant institutional review boards at participating centers, and informed consent was obtained in accordance with national regulations where applicable.

### Definitions

Acute kidney injury (AKI) was diagnosed according to the KDIGO 2012 guidelines [19].

In the retrospective cohort, the nadir value of serum creatinine during hospital admission was used as baseline serum creatinine (bsCr) [20]. Moreover, a study-specific weight-adjusted correction was applied to reduce potential AKI misclassification when baseline creatinine was estimated from nadir values (see Suppl. Info, Section A, Table S1).

In the prospective cohort, a clinical team composed by Intensive Care Unit physicians at each hospital manually validated the AKI stage diagnosed for each patient according to both the KDIGO 2012 guidelines and the clinical conditions of the patient after patient discharge from ICU.

Patients were labelled "positive" if ICU-A-AKI-2/3 occurred during ICU admission. For model validation, the time between ICU admission and the first ICU-A-AKI-2/3 trigger was used. Patients who did not develop ICU-A-AKI-2/3 were labelled "negative," with the entire ICU stay as the study period. Clinical follow-up was conducted until ICU discharge.

### Data handling

Data availability in the retrospective datasets, as a proxy of data availability in a real-world scenario, guided the selection of the initial set of clinical variables. These were harmonized across cohorts using uniform units and timestamps. To ensure data quality only physiologically plausible values were retained [21].

Data were resampled to a common hourly time grid to harmonize variables with heterogeneous sampling frequencies. For variables with multiple measurements within a given hour (e.g., vital signs), the last recorded value was retained and aligned to the subsequent full hour using a sample-and-hold approach. Urine output was aggregated over each hour and normalized by adjusted body weight.

Laboratory variables were forward-filled with a maximum carry-forward window of 4 days, while dynamic variables (e.g. vital signs) were forward-filled up to 12 h. No imputation was applied beyond these clinically defined limits.

Imputation was required to enable construction of temporal features over sliding windows.

Although XGBoost can inherently handle missing values, this preprocessing step was required to support time-dependent feature extraction. The same preprocessing pipeline was applied consistently to both retrospective and prospective datasets.

For prospective validation, the model was integrated into the production electronic health records (EHR) system of the ICU. The HL7 communication interface facilitated data exchange between the EHRs and the AI-based platform. Patient data required for model execution were automatically extracted and processed from the EHRs of all three participating hospitals as in the retrospective study. The acquisition frequency of each parameter reflects the clinical practices at each participating center. Serum creatinine and urine output measurements and other variables, were obtained as part of routine clinical care at each participating center and were not protocol-mandated by the study. Model predictions were initiated once both serum creatinine and urine output became available.

A detailed descriptions of the clinical parameters, data processing methods, and feature engineering process used in the study can be found in Suppl. Info, Section B.

### Feature selection, model training and validation

Candidate predictors were selected for their clinical relevance, guided by clinicians’ expertise and by evidence from the existing literature, as in our previous work [13]. Furthermore, a feature selection procedure using Gini importance was then applied to identify variables with the highest predictive value. The final set of predictors included vital signs (heart rate, diastolic and systolic blood pressure), laboratory tests (albumin, blood urea nitrogen (BUN), hematocrit, hemoglobin, platelet count, serum creatinine, white blood cell count), urine output. Patient demographics (age, height, weight, and gender) were collected to calculate the adjusted body weight used for urine output normalization, but were not included as independent predictors.

**Fig. 1:**
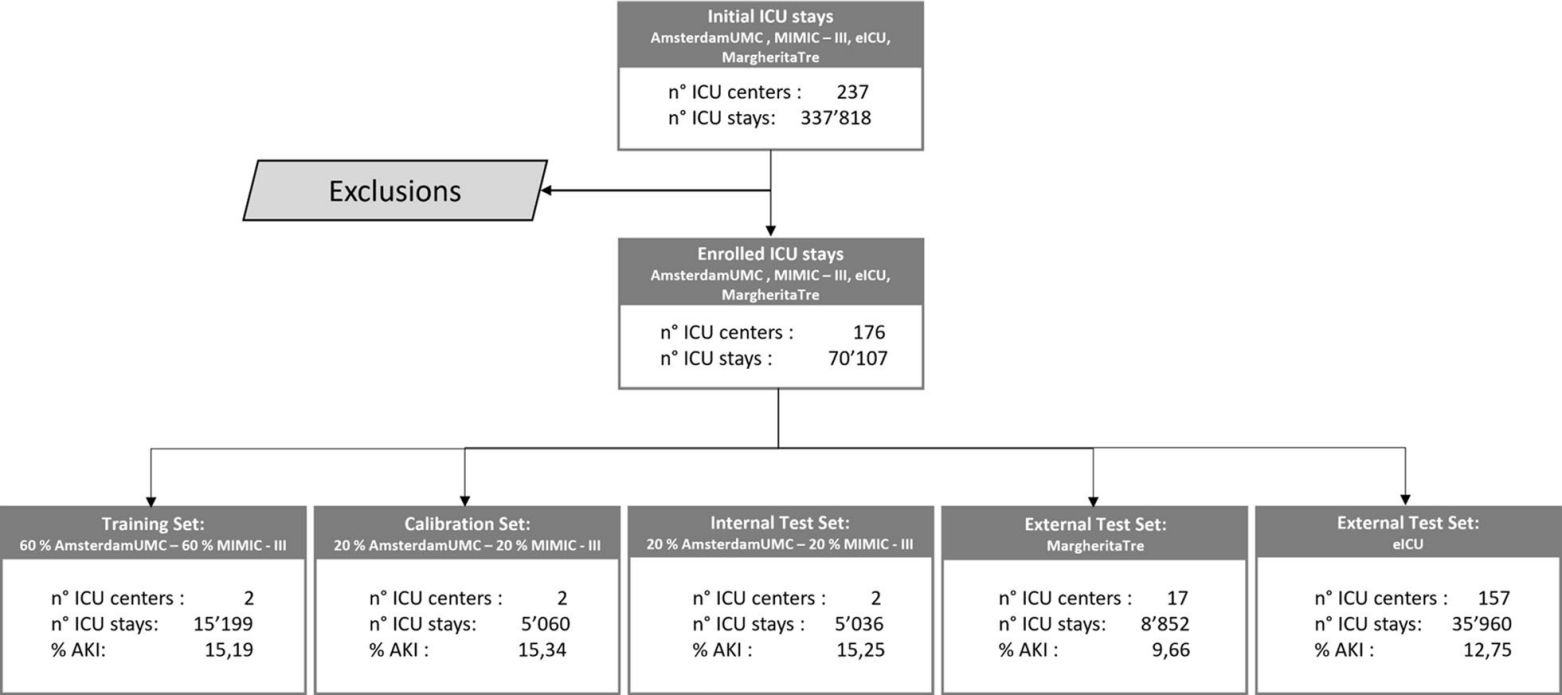
Scheme of data split into different sets. No ICU stays: Number of ICU stays associated with unique patients in each dataset

The training process was conducted using retrospective data, which were randomly split into sets as in Fig. 1. The AI model was trained on 15,199 ICU admissions from the Mimic-III and Amsterdam UMC datasets via an XGBoost classifier, which was selected because it inherently manages missing values during training by learning optimal directions for missing data at each decision tree split, effectively bypassing the need for explicit imputation [22, 23]. This allows the model to exploit any informative signal associated with missingness itself, which is particularly relevant in clinical datasets where the absence of a measurement may reflect implicit clinical decisions or patient conditions.

To optimize model performance and ensure robustness, hyperparameter tuning was performed using repeated k-fold cross-validation (k = 5) on the training set, with grid search over key XGBoost parameters, including learning rate, maximum tree depth, number of estimators, and subsampling ratios. The final configuration was selected based on average performance across folds, to ensure stability and minimize overfitting. The model calibration was then assessed on a separate calibration set to assess the reliability of the predicted probability estimates.

The model computes an hourly updated risk prediction of developing ICU-A-AKI-2/3 in the next 24 h, and the risk score is computed once at least both the serum creatinine level and the urine output are available.

Lead time was defined as the time difference between the first model prediction exceeding the predefined decision threshold and the onset of ICU-A-AKI-2/3. This metric was computed only for patients who developed AKI (true positive cases). The proportion of alarms within 24 h was defined as the percentage of true positive cases in which the model generated an alert within the 24 h preceding ICU-A-AKI-2/3 onset.

To better characterise variability in prediction timing, lead time is reported using both mean and standard deviation. Model performance (AUROC) is computed across all prediction time points, reflecting discrimination in a continuous-time prediction setting. This approach captures overall performance across heterogeneous lead times but does not explicitly stratify performance by temporal distance from AKI 2/3 onset, which should be considered when interpreting the results.

All evaluation metrics were calculated at a fixed decision threshold, determined on the calibration set. We evaluated different operating points to balance sensitivity and specificity and selected the threshold corresponding to 80% sensitivity, aiming to optimize early detection while controlling false positives (Supplementary Section D, Tables S3). The model was then validated across all populations without retraining. In the performance evaluation, each patient was counted only once, and clinicians were alerted if the risk score exceeded this threshold.

### Statistical analyses

The sample size calculation was performed specifically for the prospective validation cohort to ensure adequate precision of the AUROC estimate. It was based on an expected AUROC of 0.85 (derived from retrospective data) and an outcome prevalence of 15% resulting in a required sample size of at least 306 patients. This sample size ensures a 95% confidence interval (CI) with a margin of error of ≤ 0.10 for the AUROC estimation. No sample size constraint was applied to retrospective datasets, where all available data were used for model development and validation.

Baseline characteristics and outcomes were compared between patients with and without stage 2 or 3 AKI (KDIGO) during ICU admission. Continuous variables are presented as medians with interquartile ranges and were compared via the Mann–Whitney U test, whereas categorical variables are presented as numbers and percentages and were compared via Fisher’s exact tests (Supplementary Section E, Tables S5, S6).

Model performance was evaluated via the area under the receiver operating characteristic curve (auROC) and precision‒recall curve (auPRC). Confidence intervals for both were generated by bootstrapping (1000 replications). The sensitivity, specificity, positive predictive value (PPV), negative predictive value (NPV), positive likelihood ratio (LR+) and lead time (the time difference between the model alarm and the onset of ICU-A-AKI-2/3) were calculated at fixed thresholds.

## Results

We included 70,107 ICU admissions from 64,511 unique patients of 176 ICUs in Europe and the United States in the retrospective section of the study. The AI-based model was trained on a total of 15,199 ICU admissions derived from the Mimic-III and Amsterdam UMC datasets and then validated in the remaining cohort. In the retrospective dataset, the incidence of ICU-A-AKI-2/3 varied among various ICUs, ranging from approximately 10% to 19%. The sex distribution was 59–66% male among the cohorts, and the median age was 64–68 years. The patient characteristics are summarized in Supplementary Section C and Table S2.

In this prospective study, we enrolled 329 patients from Italy and Spain. The patient characteristics in the prospective study are described in Table 1. The incidence of ICU-A-AKI-2/3 differed across centers (9–20%). More than half of the population was male (53–65%), with a median age of 61–71 years. Statistical differences between retrospective and prospective studies were investigated and are reported in Supplementary Section E, Tables S5 and S6. In both retrospective and prospective studies, the ICU length of stay was greater for those with ICU-A-AKI-2/3. Data missingness analysis among different cohorts in retrospective and prospective studies revealed similar results (see Supplementary Section F, Tables S7 and S8).

**Table 1:** Main characteristics of the prospective enrolled population

	Maria Vittoria Hospital's cohort	Maggiore Hospital's cohort	Joan XXIII hospital's cohort
No Patients ( ≡ No ICU stays)	104	110	115
No AKI (stage 2/3 KDIGO)	10 (9.62%)	16 (14.55%)	23 (20.0%)
sCr-AKI	7(70%)	2 (12.5%)	0 (0.0%)
Uo-AKI	3(30%)	14 (87,5%)	23 (100.0%)
Gender			
Male	55 (52.88%)	72 (65.45%)	73 (63.48%)
Age			
Median	66 [55–77]	71 [57–78]	61 [48–71]
18–39	10 (10%)	12 (11%)	8 (7%)
40–49	9 (9%)	5 (5%)	20 (17%)
50–59	16 (15%)	14 (13%)	22 (19%)
60–69	25 (24%)	19 (17%)	29 (25%)
70–79	25 (24%)	34 (31%)	29 (25%)
80+	19 (18%)	26 (24%)	3 (3%)
Chronic Kidney Disease	15 (14.42%)	12 (10.91%)	0 (0%)
Reason for Admission			
Cardiovascular	7.0 (6.73%)	6.0 (5.45%)	3 (3%)
Respiratory	39.0 (37.5%)	48.0 (43.64%)	28 (24%)
Surgical	14.0 (13.46%)	16.0 (14.55%)	8 (7%)
Neurological	10.0 (9.62%)	13.0 (11.82%)	24 (21%)
Trauma	4.0 (3.85%)	0.0 (0.0%)	24 (21%)
Sepsis	9.0 (8.65%)	13.0 (11.82%)	13 (11%)
Other	42.0 (40.38%)	25.0 (22.73%)	17 (15%)
Length of ICU stay (h)	86 [44–215]	94 [48–166]	137 [70–294]

sCr-AKI: AKI defined by serum creatinine criteria; Uo-AKI: AKI defined by urine output criteria

### Retrospective cohorts

**Fig. 2:**
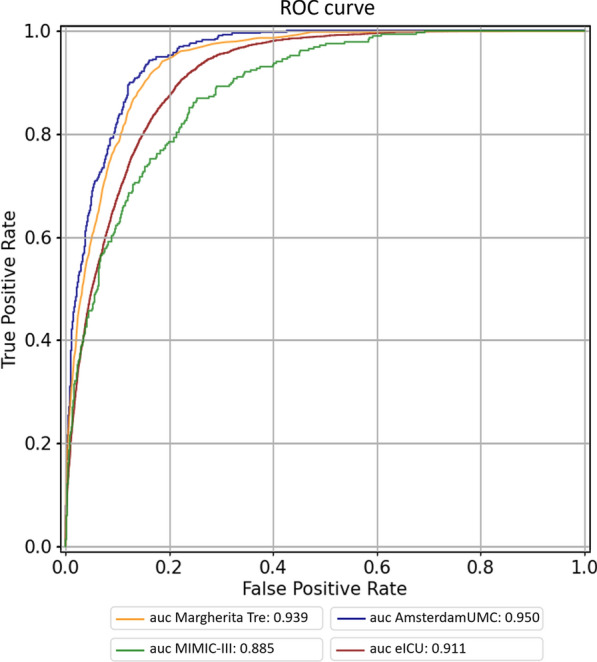
auROC curves of retrospective validation sets. auROC: area under receiver operating characteristic curve

When the model was tested on the internal test set (Amsterdam UMC and Mimic-III datasets), the auROC was 0.950 (95% CI 0.942, 0.959) for Amsterdam UMC and 0.885 (95% CI 0.867, 0.903) for Mimic-III (Fig. 2). The auPRC was 47.9% for Amsterdam UMC and 40.6% for Mimic-III. By using a fixed threshold, the model predicted the development of ICU-A-AKI-2/3 with mean lead times of 25.5 h for Amsterdam UMC and 36 h for Mimic-III. The sensitivity, specificity, and PPV are detailed in Table 2. A good balance between sensitivity and specificity was achieved in the Amsterdam UMC population, resulting in a specificity of 83.7% and a sensitivity of 89.6%. In comparison, the model applied to the Mimic-III dataset had a sensitivity of 77.2% and a specificity of 81.0%. The PPVs were close to 50% in both cohorts, likely due to the low prevalence of the positive endpoint.

**Table 2:** Model results across all the testing cohorts

Population cohorts	Design	Validation	No ICU stays	% ICU-A-AKI-2/3	auROC (CI)	auPR	Sensitivity	Specificity	Lead-Time (mean. h)	% alarms within 24 h	LR+	PPV	NPV
Mimic-III	R	I	1635	17.68	0.885 (0.867. 0.903)	0.406	0.772	0.811	36.0 + −20.4	0.762	4.08	0.467	0.943
AmsterdamUMC	R	I	3401	11.03	0.950 (0.942. 0.959)	0.479	0.837	0.896	25.5 + −19.3	0.758	8.05	0.500	0.978
eICU	R	EX	35,960	12.72	0.911 (0.907. 0.915)	0.443	0.821	0.839	23.0 + −18.2	0.772	5.10	0.426	0.970
Margherita Tre	R	EX	8852	9.65	0.939 (0.932. 0.945)	0.540	0.734	0.919	43.0 + −26.7	0.735	9.06	0.492	0.970
Maria Vittoria Hospital	P	EX	104	9.61	0.960 (0.923. 0.990)	0.448	0.968	0.700	20.4 + −15.3	0.833	3.23	0.700	0.968
Maggiore Hospital	P	EX	110	14.55	0.869 (0.802. 0.943)	0.355	0.798	0.750	14.0 + −12.2	0.778	3.19	0.387	0.949
Joan XXIII Hospital	P	EX	115	20.00	0.820 (0.733. 0.916)	0.428	0.761	0.826	17.6 + −13.0	0.712	4.37	0.463	0.946

R: Retrospective study. P: Prospective study. PPV: positive predictive value. NPV: negative predictive value. LR+: Likelihood ratio positive. auROC: area under the receiver operating characteristic curve. auPR: area under the precision recall curve; % alarms within 24 h: proportion of true positive cases with the model alert occurring within 24 h before AKI onset, CI: Confidence Interval. I: Internal Test set. EX: External Test Set

In the external retrospective validation, the auROC was 0.911 (95% CI 0.907, 0.915) for the eICU and 0.939 (95% CI 0.932, 0.945) for Margherita Tre (Fig. 2). The specificity and sensitivity were 83.9% and 82.1%, respectively, for the eICU and 91.9% and 73.4%, respectively, for Margherita Tre. Additionally, mean lead times ranged from 23 h in the eICU to 43 h in Margherita Tre [13]. Notably, over 73.5% of true positive alerts occurred within the 24 h preceding AKI-2/3 onset, indicating that most predictions were generated within a clinically actionable timeframe.

Details of the comparison between the previously developed and enhanced AI models are provided in Supplementary Section G and Table S9.

To ensure the model's fairness and generalizability, we conducted additional tests to identify and solve potential biases in the retrospective population. Bias checks across key demographic and clinical subgroups, including age and comorbidities. These analyses revealed minimal to no significant bias, supporting the robustness of the model in heterogeneous ICU populations. Details of these assessments are reported in Supplementary Section H and Figs. S1–S4.

In addition, to evaluation the model's performance and reliability, we computed Brier Score, as it reflects the model's calibration across cohorts. The Brier Score was 0.058 on the AmsterdamUMC test set, 0.099 on the Mimic-III test set, and 0.079 on the eICU test set.

### Prospective cohorts

In the prospective cohort, the first model prediction occurred after a mean of 9.0 ± 6.5 from ICU admission, corresponding to the availability of both serum creatinine and urine output. (Supplementary Section K and Table S15), while the time from ICU admission to AKI onset is reported in Supplementary Section J (Table S14).

**Fig. 3:**
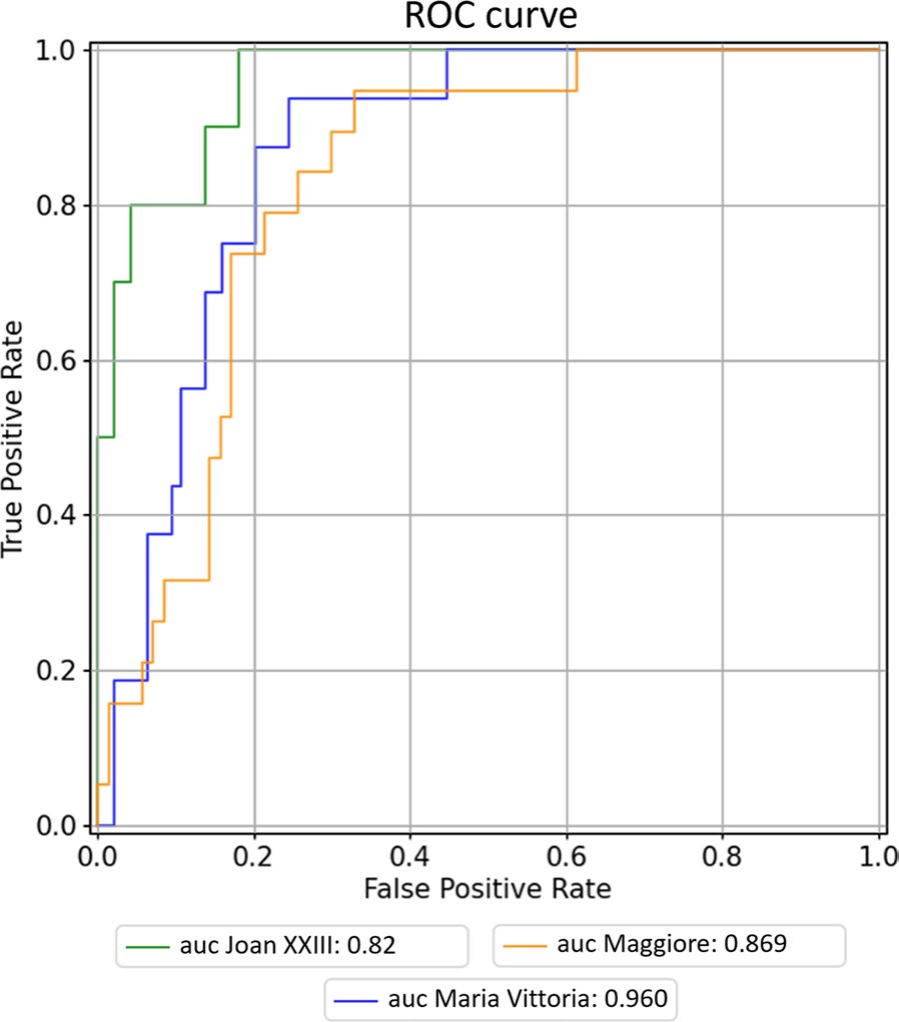
auROC curves of prospective validation sets. auROC: Area under receiver operating characteristic curve

The AI model tested on 104 patients at Maria Vittoria achieved an auROC of 0.960 (95% CI 0.92, 0.99), with a sensitivity of 70%, specificity of 97%, and mean lead time of 20 h at the preselected threshold determined during the development phase of the model. At Maggiore Hospital, with 110 enrolled patients, the model achieved an auROC of 0.869 (95% CI, 0.80, 0.94), a sensitivity of 75%, and a specificity of 80%, with a mean lead time of 14 h. When the model was tested on 115 patients from Joan XXIII Hospital, the auROC was 0.820 (95% CI, 0.73, 0.92), with a sensitivity and specificity of 76% and 83%, respectively. The mean lead time was 18 h (Fig. 3). The AuPRC was equal to 0.448, 0.355, and 0.428 in Maria Vittoria, Maggiore, and Joan XXIII Hospital, respectively. Additionally, the computed PPVs were 70%, 39%, and 46%, respectively. In the pooled cohort of 329 patients, the AI model achieved an auROC of 0.876 (95% CI, 0.836, 0.926), a sensitivity of 77%, a specificity of 84%, and a mean lead time of 17 h (Table 2). The distribution of lead times showed variability across patients, with both short-term and longer-term predictions. Over 70% of true positive alerts occurred within the 24 h preceding ICU-A-AKI-2/3 onset, indicating that most predictions were generated within a clinically actionable timeframe.

We also assessed the model’s calibration at each individual center where the prospective study was conducted by computing the Brier Score, it was 0.057 for Maria Vittoria Hospital, 0.106 for Maggiore Hospital, and 0.135 for Joan XXIII Hospital.

### Sensitivity analysis

As described in the methods, owing to frequent missing sCr measurements before ICU admission, we used the nadir in-hospital sCr value as bSCr in the retrospective study.

A sensitivity analysis was conducted by changing the bSCr calculation methods to test the model's robustness. The AI model maintained strong predictive accuracy for ICU-A-AKI-2/3 across different bSCr methods, with auROCs > 0.82 in all cohorts. Further details are provided in Supplementary Section I, Tables S10 and S11.

## Discussion

We developed and extensively validated an improved AI model for predicting ICU-A-AKI-2/3, with retrospective validation and prospective validation across multiple centers. The model uses only existing clinical variables and adapts to real-world ICU scenarios by handling missing data.

In all settings, it demonstrated the ability to predict ICU-A-AKI-2/3 risk with high sensitivity and specificity, showing promising results in terms of auROCs (0.82–0.96) over multiple ICUs in various countries. Comprehensive validation, including internal, external, and prospective assessments, highlights the model's robust performance, which is rare among AI models for ICU AKI prediction [24]. The developed model is intended as a clinical decision support tool. When a patient is flagged as high-risk, clinicians may be prompted to implement preventive strategies aligned with KDIGO recommendations, including optimization of hemodynamic status and perfusion pressure, review and discontinuation of nephrotoxic agents, adjustment of drug dosing based on estimated glomerular filtration rate, and closer monitoring of renal function and urine output.

Lead times on the order of several hours to one day are generally sufficient to enable these interventions in ICU practice, supporting the clinical relevance of the model predictions.

The moderate PPV values are expected given the relatively low event prevalence and the selected operating point, which prioritizes sensitivity and early detection. For this reason, the model is intended to support clinical reassessment rather than trigger an automatic intervention.

In contrast to other predictive approaches, our model offers distinct advantages. Liang et al*.* developed and prospectively validated a model for the prediction of severe AKI 48 h post-ICU admission, achieving values of 0.86 (retrospective) and 0.84 (prospective) [25]. However, its reliance on clinical notes, daily risk scores, and single-center validation limits its generalizability.

Sun et al. developed and prospectively validated AI models for predicting various clinical complications, including AKI, in ICUs and hospital wards [26, 27]. However, their model differs from ours in that it is not intensive care unit (ICU) specific, lacks continuous predictions, and provides results only at admission and discharge. It requires recalibration for each hospital, which may limit its scalability and deployment across diverse locations.

Flechet et al*.* prospectively validated an AKI predictor, a machine-learning model, to predict AKI 2/3 in ICU patients and compared the results to clinician judgment [28]. The model performed similarly to physicians in predicting both oliguric and nonoliguric AKI-2/3, despite being trained specifically for creatinine-based AKI. However, its predictions were limited to ICU admission and 24 h later, it lacked continuous monitoring, and it required manual data entry because of the absence of EHR integration.

The other approach to AKI prediction and patient risk stratification in the ICU is the use of biomarkers. Several biomarkers, including NGAL, TIMP-2, IGFBP7 and L-FABP, have been proposed in recent years [29]. Although promising, their integration into clinical practice faces challenges, including varying thresholds found across studies, biases derived from acute and chronic health conditions, and difficulties in determining optimal testing times owing to the ambiguity of early kidney deterioration signs.

Unlike biomarkers, our model operates automatically without requiring additional blood or urine sampling. It provides hourly risk predictions, allowing clinicians to monitor risk score trajectories and detect both oliguric and nonoliguric AKI associated with various etiologies [4, 30].

Prospective multicenter validation confirmed the robustness of our AI model across different ICU settings without requiring recalibration. Despite a slight performance drop from retrospective to prospective validation, the model maintained good to excellent discrimination (auROCs > 0.82) and a mean lead time ranging from 14 to 20 h across datasets, demonstrating its effectiveness in real-world clinical environments for early AKI detection and management.

The reported lead time of 14–20 h refers to mean values across datasets and does not represent a fixed prediction horizon. In this continuous prediction framework, risk is dynamically updated over time, and lead time emerges from the timing of threshold crossing. Model discrimination (AUROC) is computed across all prediction time points and therefore reflects aggregate performance over heterogeneous lead times. Differences in mean lead time across datasets likely reflect differences in data density and clinical trajectories, and should be considered when interpreting cross-dataset comparisons.

In particular, we observed a relevant difference in the prevalence of ICU-acquired AKI stage 2/3 across centers, ranging from 9 to 20%, as patients were enrolled in general ICUs across different countries, where differences in AKI incidence and CKD prevalence likely reflect variation in case mix, illness severity, and patient populations. This heterogeneity may influence both model calibration and discrimination, as models trained on aggregated data can be sensitive to shifts in outcome prevalence and variable distributions, especially in critical care contexts where local protocols and resource availability differ substantially [16]. Notably, Maria Vittoria Hospital achieved the highest auROC and lead-time, likely due to its greater similarity to the training cohorts and potentially lower ICU complication rates, as suggested by shorter average ICU length of stay.

The strong performance observed in our study may be attributed to several methodological factors, including the use of high-resolution longitudinal data, rigorous data harmonization across heterogeneous datasets, explicit modeling of temporal dynamics through sliding-window features, and extensive external and prospective validation across centers.A minor variation in Brier Scores across centers was observed, reflecting small differences in calibration performance. The highest score (0.135) was observed at Joan XXIII Hospital, which may relate to a higher AKI prevalence and differences in cohort characteristics compared to the training population, factors known to influence the alignment between predicted and observed risks. Importantly, these variations remain within an acceptable range and are not indicative of reduced generalizability, but rather reflect the expected challenges of applying predictive models across heterogeneous clinical settings.

The 27th Acute Disease Quality Initiative (ADQI) international workgroup highlighted the need for digital tools to enhance AKI management in the ICU [31]. These tools can help identify high-risk patients for tailored surveillance and interventions. Interventional trials have shown that a KDIGO prevention bundle significantly reduces moderate and severe AKI incidence, underscoring the potential of timely risk stratification [32, 33]. Our AI model aligns with this vision, offering continuous risk monitoring and supporting timely interventions, such as KDIGO bundle implementation. Future studies could explore the integration of AI models with biomarkers to leverage their combined strengths, potentially refining risk stratification, reducing unnecessary testing, and enhancing clinical decision-making.

## Limitations

Our study has several potential limitations. First, we excluded pediatric patients, so our model cannot be applied to this population, despite its potential benefits, as recently suggested by Fragasso et al. [34].

Second, the prospective validation was observational and did not include active interventions, so while the model’s performance was assessed, its impact on clinical outcomes remains to be determined. Future research should focus on integrating the AI model into ICU workflows and AKI care bundles to assess its effect on clinical and economic outcomes.

Third, the lack of measured bSCr values requires estimation for patients. In this retrospective study, we used the nadir in-hospital sCr value. Siew et al. [20] found that using nadir in-hospital sCr achieved 81.7% sensitivity and 79.8% specificity for AKI diagnosis compared with pre-hospital baseline values. This method caused some misclassifications: ~ 2.8% for stage 2/3 AKI and ~ 1.5% for the negative endpoint, similar to the findings of Za'vada et al*.* [35]. Notably, Siew et al*.* [20] did not include the urine output criterion, which we incorporated to reduce sCr-related inaccuracies. Sensitivity analysis confirmed the AI model's robustness despite differing bSCr estimation methods in retrospective and prospective validations. However, slightly lower performance was observed when nadir bSCr was used, as this method may overestimate AKI incidence owing to fluid therapy-related sCr dilution, especially in smaller samples.

A further limitation relates to the use of multicenter datasets for the development phase, which may introduce unrecognized biases or heterogeneity since not designed specifically for training predictive modeling. Although we implemented extensive data harmonization aligning units of measurement, outlier removal, data formatting across centers to ensure consistency in how input features were represented and conducted subgroup bias analyses to minimize these effects, residual confounding cannot be fully excluded. Future larger prospective validation studies should continue to monitor and address potential biases in diverse patient populations.

An additional limitation relates to the exclusion of patients with missing serum creatinine or urine output data required for AKI definition. These exclusions were applied prior to analysis and may introduce selection bias, as excluded patients could differ systematically from the analyzed population in terms of severity of illness, monitoring intensity, or clinical trajectory. Consequently, the reported incidence of AKI and model performance may not fully reflect populations with more limited data availability.

## Conclusions

The results of this study are encouraging and indicate the feasibility of using our AI model to predict moderate and severe AKI in ICU patients by leveraging real-time electronic health record data. The model demonstrated robust prospective performance across several metrics, including accuracy, sensitivity, specificity, likelihood ratios, positive predictive value, and lead time.

Although the model demonstrated strong discriminative performance and prospective validation, observational evaluation alone does not establish its impact on clinical outcomes. The effectiveness of a prediction model depends on its integration into clinical workflows and the actions it triggers. Therefore, interventional studies (e.g., pragmatic or stepped-wedge trials) are needed to assess whether model-guided care improves outcomes and to evaluate potential unintended effects.

## Software specification

Statistical analyses and data processing were performed via Python (version 3.7.9; Python Software Foundation: http://www.python.org). The XGBoost model was implemented with the scikit-learn library in Python.

## Supplementary Information

Supplementary material 1.

## Data Availability

Deidentified patient data from the Medical Information Mart for Intensive Care III (Mimic-III) v1.3 database are publicly available at https://mimic.mit.edu/. Deidentified patient data from the AmsterdamUMCdb (AmsterdamUMC) are publicly available at https://amsterdammedicaldatascience.nl/#amsterdamumcdb. Deidentified patient data from the eICU Collaborative Research Database (eICU) are publicly available at https://eicu-crd.mit.edu/. The Margherita Tre dataset is available upon request, as there are ethical restrictions on sharing data publicly, and the data are owned by a third-party organization. Agreement on data sharing has to be requested from Istituto di Ricerche Farmacologiche Mario Negri (giviti@marionegri.it) and evaluated by an ethical committee. The datasets generated during the prospective clinical study were collected through the software system implementing the study model. Due to patient privacy and institutional regulations, the data are not publicly available but may be made available from the corresponding author upon reasonable request and subject to appropriate approval.

## Abbreviations

AKI: Acute kidney injury
auPR: Area under the precision-recall curve
auROC: Area under the receiver operating characteristic curve
bSCr: Basal value of serum creatinine
CA-AKI: Community-acquired AKI
EHR: Electronic health record
ICU-A-AKI: ICU-acquired AKI
ICU: Intensive care unit
IGFBP7: Insulin-like growth factor binding protein 7
KDIGO: Kidney disease: improving global outcomes
NGAL: Neutrophil gelatinase-associated lipocalin
sCr: Serum creatinine
sCr-AKI: Acute kidney injury detected by applying the serum creatinine criterion
TIMP-2: Tissue inhibitor of metalloproteinases-2
UO: Urine output
uo-AKI: Acute kidney injury detected by applying urine output criteria
