# Supplementary material for: Prospective and external evaluation of an AI model for continuous and early prediction of moderate and severe AKI in critically ill patients
Source: Intensive Care Med Exp. 2026 Jun 10;14:71. doi: 10.1186/s40635-026-00928-y (PMC13253924; doi:10.1186/s40635-026-00928-y)
Supplement: Supplementary file 1 — Supplementary material 1. [file 40635_2026_928_MOESM1_ESM.docx]

# Prospective and external evaluation of an AI model for continuous and early prediction of moderate and severe AKI in critically ill patients

## SUPPLEMENTARY INFO

### Definition of Acute Kidney Injury in the retrospective cohort: A patient’s weight adjusted strategy

In the retrospective analysis, the nadir values along the entire ICU stay (before AKI onset) were used to estimate the baseline serum creatinine (bsCr) for each patient in the retrospective cohort [1]. However, the AKI severity stage identification in these patients could be inaccurate due to potential imprecise bsCr values due to data-entry errors or factors such as prolonged hospitalization, which may lead to muscle mass loss and falsely low serum creatinine values. To account for both possible reasons of inaccurate bsCr values, we adjusted AKI staging based on the patient's admission weight and measured creatinine value at the time of admission, indirectly accounting for the impact of ICU length of stay on creatinine levels. To address the possible misclassification of ICU-A-AKI due to lower baseline estimates, we modified the severity stage of AKI, as detailed in **Table S1**, by adjusting the stage to a lower level.

Table S1: Weight-Adjusted strategy to assign KDIGO stage based on serum creatinine.

| **Initial AKI Stage** | **Condition** | **Final AKI Stage** |
| --- | --- | --- |
| 3 | weight < 50Kg & sCr < 1.8 mg/dL | 2 |
| 3 | 50kg ≤ weight < 90Kg & sCr < 2.1 mg/dL | 2 |
| 3 | weight ≥ 90 & sCr < 2.85 mg/dL | 2 |
| 2 | weight < 50Kg & sCr < 1.2 mg/dL | 1 |
| 2 | 50kg ≤ weight < 90Kg & sCr < 1.4 mg/dL | 1 |
| 2 | weight ≥ 90 & sCr < 1.9 mg/dL | 1 |

### Data Processing: sample and hold strategy for time series reconstruction and feature engineering

After extracting, normalizing, and cleaning the raw clinical data, time series were created with 1-hour time steps for both the retrospective and prospective validations. Missingness was variable-dependent and reflected the heterogeneous sampling frequencies typical of ICU data, with vital signs being more frequently recorded and laboratory measurements more sparse.. For variables with multiple measurements within a 1-hour window, such as heart rate and systolic/diastolic blood pressure, the last recorded value within the window was used and assigned to the next full hour. For urine output, measurements within the time window were summed, converted to an hourly rate, and normalized by the patient’s adjusted body weight, following clinical practice for evaluating urine output trends. If no measurements were available for more than 12 hours, no imputation was performed. These constraints were defined to ensure clinical plausibility and to avoid propagating outdated values.

The adjusted body weight was calculated based on the patient’s weight at ICU admission. Patients for whom the admission weight was unavailable were excluded from the analysis.

For laboratory data (albumin, blood urea nitrogen (BUN), hematocrit, hemoglobin, platelet count, serum creatinine, white blood cell count), derived from blood tests or blood gas analysis, a "fill-forward" strategy was applied, ensuring that values were not carried forward for more than 4 days. All imputations were performed in a strictly forward manner (sample-and-hold), ensuring that only past information was used at each time point and preventing any information leakage from future observations.

To feed the AI model, features derived from clinical variables were computed using sliding windows.

Laboratory trends were assessed using window sizes of 12, 18, 24, 36, 48, and 72 hours, while dynamic vital signs (e.g., heart rate, blood pressure) were assessed using window sizes of 6, 12, 18, 24, 36, and 48 hours.

The same preprocessing and imputation pipeline was applied consistently to both retrospective and prospective datasets.

### Patients’ characteristics of the retrospective cohort

*Table S2: Main characteristics of the retrospective enrolled population*

|  | **AmsterdamUMC** | | **eICU** | | **Mimic-III** | | **Margherita-Tre** | |
| --- | --- | --- | --- | --- | --- | --- | --- | --- |
|  | value | % or [iqr] | value | % or [iqr] | value | % or [iqr] | value | % or [iqr] |
| ***n° ICU centres*** | 1 |  | 157 |  | 1 |  | 17 |  |
| ***n° ICU stays*** | 17142 |  | 35960 |  | 8153 |  | 8852 |  |
| ***n° Patients*** | 15589 |  | 32678 |  | 7730 |  | 8514 |  |
| ***n° AKI (stage 2/3 KDIGO)*** | 1875 | 10,94 | 4574 | 12,72 | 1447 | 17,75 | 855 | 9,66 |
| *sCr-AKI* | 505 | 26,93 | 769 | 16,81 | 403 | 27,85 | 328 | 38,36 |
| *Uo-AKI* | 1356 | 72,32 | 3792 | 82,90 | 1033 | 71,39 | 523 | 61,17 |
| *Uo-sCr-AKI* | 14 | 0,75 | 13 | 0,28 | 11 | 0,76 | 4 | 0,47 |
| ***Median onset of AKI stage 2/3 KDIGO***  ***(hours from ICU admission)*** | 34 | [22.0, 53.0] | 34 | [23.0, 57.0] | 38 | [24.0, 74.0] | 41 | [26.0,  89.0] |
| ***Gender*** |  |  |  |  |  |  |  |  |
| *Male* | 11375 | 66,36 | 20468 | 56,92 | 5025 | 61,63 | 5342 | 60,35 |
| ***Age*** |  |  |  |  |  |  |  |  |
| *median* | 65 | [55.0, 75.0] | 64 | [52.0, 75.0] | 66,23 | [54.73, 76.59] | 67 | [55.0, 75.01] |
| *18-39* | 1628 | 9,50 | 4047 | 11,25 | 551 | 6,76 | 722 | 8,16 |
| *40-49* | 1490 | 8,69 | 3459 | 9,62 | 768 | 9,42 | 785 | 8,87 |
| *50-59* | 2899 | 16,91 | 6726 | 18,70 | 1465 | 17,97 | 1479 | 16,71 |
| *60-69* | 4748 | 27,70 | 8122 | 22,59 | 1819 | 22,31 | 2126 | 24,02 |
| *70-79* | 4732 | 27,60 | 7465 | 20,76 | 2041 | 25,03 | 2602 | 29,39 |
| *80+* | 1645 | 9,60 | 6141 | 17,08 | 1509 | 18,51 | 1138 | 12,86 |
| ***Ethnicity*** |  |  |  |  |  |  |  |  |
| *African American* | - | - | 3375 | 9,39 | 383 | 4,70 | - | - |
| *Caucasian* | - | - | 28949 | 80,50 | 5524 | 67,75 | - | - |
| *Hispanic* | - | - | 1765 | 4,91 | 183 | 2,24 | - | - |
| *Native American* | - | - | 183 | 0,51 | 4 | 0,05 | - | - |
| *Asian* | - | - | 352 | 0,98 | 155 | 1,90 | - | - |
| *Other/Unknown* | 15589 | 100 | 1239 | 3,45 | 1904 | 23,35 | 8852 | 100 |
| ***Comorbidity*** |  |  |  |  |  |  |  |  |
| *Cancer* | - | - | 2588 | 7,20 | 685 | 8,40 | 989 | 11,17 |
| *Chronic Kidney Disease* | - | - | 3784 | 10,52 | 330 | 4,05 | 430 | 4,86 |
| *Congestive Heart Failure* | - | - | 3112 | 8,65 | 2297 | 28,17 | 1404 | 15,86 |
| *Coronary Artery Disease* | - | - | 2145 | 5,96 | 3718 | 45,60 | 777 | 8,78 |
| *COPD* | - | - | 3020 | 8,40 | 1445 | 17,72 | 728 | 8,22 |
| *Hypertension* | - | - | 6357 | 17,68 | 4494 | 55,12 | 4528 | 51,15 |
| *Diabetes Mellitus* | - | - | 7507 | 20,88 | 2093 | 25,67 | 1393 | 15,74 |
| ***ICU admission Type*** |  |  |  |  |  |  |  |  |
| *Coronary Care/CardioToracic* | - | - | 4192 | 11,66 | 1256 | 15,41 | - | - |
| Trauma Surgical | - | - | - | - | 736 | 9,03 | - | - |
| *Medical-Surgical* | - | - | 20035 | 55,71 | - | - | - | - |
| *Neurological* | - | - | 2834 | 7,88 | - | - | 1163 | 13,14 |
| *Medical* | - | - | 2232 | 6,21 | 1256 | 15,41 | 4642 | 52,44 |
| *Surgical* | - | - | 2797 | 7,78 | 1083 | 13,28 | - | - |
| *Cardiac* | - | - | 2694 | 7,49 | - | - | 2264 | 25,58 |
| *Cardio - Surgical* | - | - | 1176 | 3,27 | 3822 | 46,88 | 783 | 8,85 |
| *Intensive Care* | 12462 | 72,70 | - | - | - | - | - | - |
| *Medium Care* | 3094 | 18,05 | - | - | - | - | - | - |
| *Intensive Care & Medium Care* | 1586 | 9,25 | - | - | - | - | - | - |
| *Other* | - | - | - | - | - | - | - | - |
| ***Reason for ICU admission*** |  |  |  |  |  |  |  |  |
| *Cardiovascular* | 617 | 3,60 | 9347 | 25,99 | 1957 | 24,00 | 2384 | 26,93 |
| *Neurological* | 439 | 2,56 | 2684 | 7,46 | 431 | 5,29 | 2100 | 23,72 |
| *Respiratory* | 1110 | 6,48 | 4910 | 13,65 | 521 | 6,39 | 1349 | 15,24 |
| *Surgical* | 8418 | 49,11 | 6327 | 17,59 | 2521 | 30,92 | 1596 | 18,03 |
| *Sepsis* | 1829 | 10,67 | 3471 | 9,65 | 124 | 1,52 | 278 | 3,14 |
| *Trauma* | 998 | 5,82 | 4093 | 11,38 | 287 | 3,52 | 1486 | 16,79 |
| *Other* | 4945 | 28,85 | 9029 | 25,11 | 1833 | 22,48 | - | - |
| ***RRT*** | 365 | 2,13 | 410 | 1,14 | 137 | 1,68 | 22 | 0,25 |
| ***Baseline SCr (mg/dL)*** | 0,77 | [0.6, 0.97] | 0,72 | [0.56, 0.94] | 0,70 | [0.5, 0.9] | 0,71 | [0.58,0.89] |
| ***Length of ICU stay (h)*** | 25,37 | [20.87, 79.99] | 41,48 | [24.27, 71.68] | 58,00 | [31.82, 117.23] | 34,74 | [20.02, 92.52] |
| ***In-ICU mortality*** | 1129 | 6,59 | 1417 | 3,94 | 2822 | 34,61 | 605 | 6,83 |

*Legend: COPD: Chronic Obstructive Pulmonary Disease, RRT: Renal Replacement Therapy,sCr-AKI: AKI defined by serum creatinine criteria; Uo-AKI: AKI defined by urine output criteria*

1. **Selection of the Decision Threshold**

The selection of a decision threshold was necessary to enable the AI model to generate real-time alerts for predict the risk of AKI stage 2-3 KDIGO. The threshold defines the operating point that balances early detection and false-positive rate. To identify the optimal cut-off, the model was evaluated on the **Calibration Set,** which included 5,060 ICU stays from AmsterdamUMC and MIMIC-III. Sensitivity was progressively varied between 60% and 95% in 5% increments, and the corresponding thresholds were computed to assess model performance at different operating points.

The **default threshold** was defined as the one corresponding to **80% sensitivity** on the Calibration Set, providing **86% specificity**, and an estimated **precision of 48% (Table S3).**

The selected threshold was then applied to both the **internal** and the **external validation cohorts** to confirm generalizability across healthcare systems. Across all datasets, the AI model maintained **auROC values above 0.90,** with **average sensitivity ≈ 79%, specificity ≈ 88%,** and **precision ≈ 48%.**

Performances at alternative thresholds are reported in **Table S4.**

Table S3: AI-model performances on the Calibration set at varying sensitivity levels.

| ***Dataset*** | ***n° Stays*** | ***% AKI (stage 2/3 KDIGO)*** | ***auROC*** | ***Fixed Sensitivity*** | ***Threshold*** | ***PPV*** | ***NPV*** |
| --- | --- | --- | --- | --- | --- | --- | --- |
| Calibration Set | 5060 | 15,34 | 0.924 | 60% | 0,409 | 0,607 | 0,939 |
|  |  |  |  | 65% | 0,374 | 0,560 | 0,945 |
|  |  |  |  | 70% | 0,343 | 0,530 | 0,952 |
|  |  |  |  | 75% | 0,315 | 0,505 | 0,959 |
|  |  |  |  | **80%** | **0,288** | **0,478** | **0,966** |
|  |  |  |  | 85% | 0,259 | 0,451 | 0,974 |
|  |  |  |  | 90% | 0,226 | 0,416 | 0,981 |
|  |  |  |  | 95% | 0,182 | 0,364 | 0,990 |

Legend: R: Retrospective study. P: Prospective study. PPV: positive predictive value. NPV: negative predictive value; auROC: area under the receiver operating characteristic curve.

Table S4: AI-model performances when using different thresholds over testing populations.

| ***Dataset*** | ***n° Stays*** | ***% AKI (stage 2/3 KDIGO)*** | ***auROC*** | ***Fixed Sensitivity (on Calibration Set)*** | ***Threshold*** | ***Sensitivity*** | ***Specificity*** | ***PPV*** | ***NPV*** |
| --- | --- | --- | --- | --- | --- | --- | --- | --- | --- |
| Internal Test | 5036 | 15,34 | 0,929 | 60% | 0,409 | 0,637 | 0,941 | 0,621 | 0,945 |
| External Test (eICU) | 35960 | 12,75 | 0,911 | 60% | 0,409 | 0,565 | 0,931 | 0,545 | 0,936 |
| External Test (Margherita Tre) | 8852 | 9,66 | 0, 911 | 60% | 0,409 | 0,505 | 0,978 | 0,625 | 0,948 |
| Averaged Results | - | - | - | 60% | 0,409 | 0,569 | 0,950 | 0,597 | 0,943 |
|  |  |  |  |  |  |  |  |  |  |
| Internal Test | 5036 | 15,34 | 0,929 | 65% | 0,374 | 0,681 | 0,923 | 0,573 | 0,950 |
| External Test (eICU) | 35960 | 12,75 | 0,911 | 65% | 0,374 | 0,643 | 0,911 | 0,513 | 0,946 |
| External Test (Margherita Tre) | 8852 | 9,66 | 0,939 | 65% | 0,374 | 0,643 | 0,957 | 0,858 | 0,953 |
| Averaged Results | - | - | - | - | - | 0,656 | 0,930 | 0,648 | 0,950 |
|  |  |  |  |  |  |  |  |  |  |
| Internal Test | 5036 | 15,34 | 0,929 | 70% | 0,343 | 0,729 | 0,906 | 0,540 | 0,957 |
| External Test (eICU) | 35960 | 12,75 | 0,911 | 70% | 0,343 | 0,708 | 0,889 | 0,481 | 0,954 |
| External Test (Margherita Tre) | 8852 | 9,66 | 0,939 | 70% | 0,343 | 0,611 | 0,946 | 0,548 | 0,958 |
| Averaged Results | - | - | - | - | - | 0,683 | 0,914 | 0,523 | 0,956 |
|  |  |  |  |  |  |  |  |  |  |
| Internal Test | 5036 | 15,34 | 0,929 | 75% | 0,315 | 0,768 | 0,890 | 0,515 | 0,962 |
| External Test (eICU) | 35960 | 12,75 | 0,911 | 75% | 0,315 | 0,771 | 0,865 | 0,453 | 0,963 |
| External Test (Margherita Tre) | 8852 | 9,66 | 0,939 | 75% | 0,315 | 0,673 | 0,932 | 0,515 | 0,963 |
| Averaged Results | - | - | - | - | - | 0,737 | 0,896 | 0,494 | 0,963 |
|  |  |  |  |  |  |  |  |  |  |
| **Internal Test** | **5036** | **15,34** | **0,929** | **80%** | **0,288** | **0,809** | **0,870** | **0,486** | **0,968** |
| **External Test (eICU)** | **35960** | **12,75** | **0,911** | **80%** | **0,288** | **0,821** | **0,839** | **0,426** | **0,970** |
| **External Test (Margherita Tre)** | **8852** | **9,66** | **0,939** | **80%** | **0,288** | **0,734** | **0,919** | **0,492** | **0,970** |
| **Averaged Results** | **-** | **-** | **-** | **-** | **-** | **0,788** | **0,876** | **0,468** | **0,969** |
|  |  |  |  |  |  |  |  |  |  |
| Internal Test | 5036 | 15,34 | 0,929 | 85% | 0,259 | 0,866 | 0,843 | 0,456 | 0,976 |
| External Test (eICU) | 35960 | 12,75 | 0,911 | 85% | 0,259 | 0,864 | 0,808 | 0,396 | 0,976 |
| External Test (Margherita Tre) | 8852 | 9,66 | 0,939 | 85% | 0,259 | 0,777 | 0,903 | 0,461 | 0,974 |
| Averaged Results | - | - | - | - | - | 0,836 | 0,851 | 0,438 | 0,975 |
|  |  |  |  |  |  |  |  |  |  |
| Internal Test | 5036 | 15,34 | 0,929 | 90% | 0,226 | 0,902 | 0,809 | 0,418 | 0,982 |
| External Test (eICU) | 35960 | 12,75 | 0,911 | 90% | 0,226 | 0,913 | 0,766 | 0,362 | 0,984 |
| External Test (Margherita Tre) | 8852 | 9,66 | 0,939 | 90% | 0,226 | 0,843 | 0,879 | 0,427 | 0,981 |
| Averaged Results | - | - | - | - | - | 0,886 | 0,818 | 0,402 | 0,982 |
|  |  |  |  |  |  |  |  |  |  |
| Internal Test | 5036 | 15,34 | 0,929 | 95% | 0,182 | 0,940 | 0,748 | 0,362 | 0,988 |
| External Test (eICU) | 35960 | 12,75 | 0,911 | 95% | 0,182 | 0,957 | 0,694 | 0,313 | 0,991 |
| External Test (Margherita Tre) | 8852 | 9,66 | 0,939 | 95% | 0,182 | 0,920 | 0,832 | 0,369 | 0,989 |
| Averaged Results | - | - | - | - | - | 0,939 | 0,758 | 0,348 | 0,989 |

Legend: R: Retrospective study. P: Prospective study. PPV: positive predictive value. NPV: negative predictive value; auROC: area under the receiver operating characteristic curve.

### Statistical differences between-groups

Table S5: A between-group comparison of subgroups of patient with and without in-ICU-acquired AKI stage 2/3.

Table S6: A between-group comparison of subgroups of patient with and without in-ICU-acquired AKI stage 2/3 for retrospective cohorts.


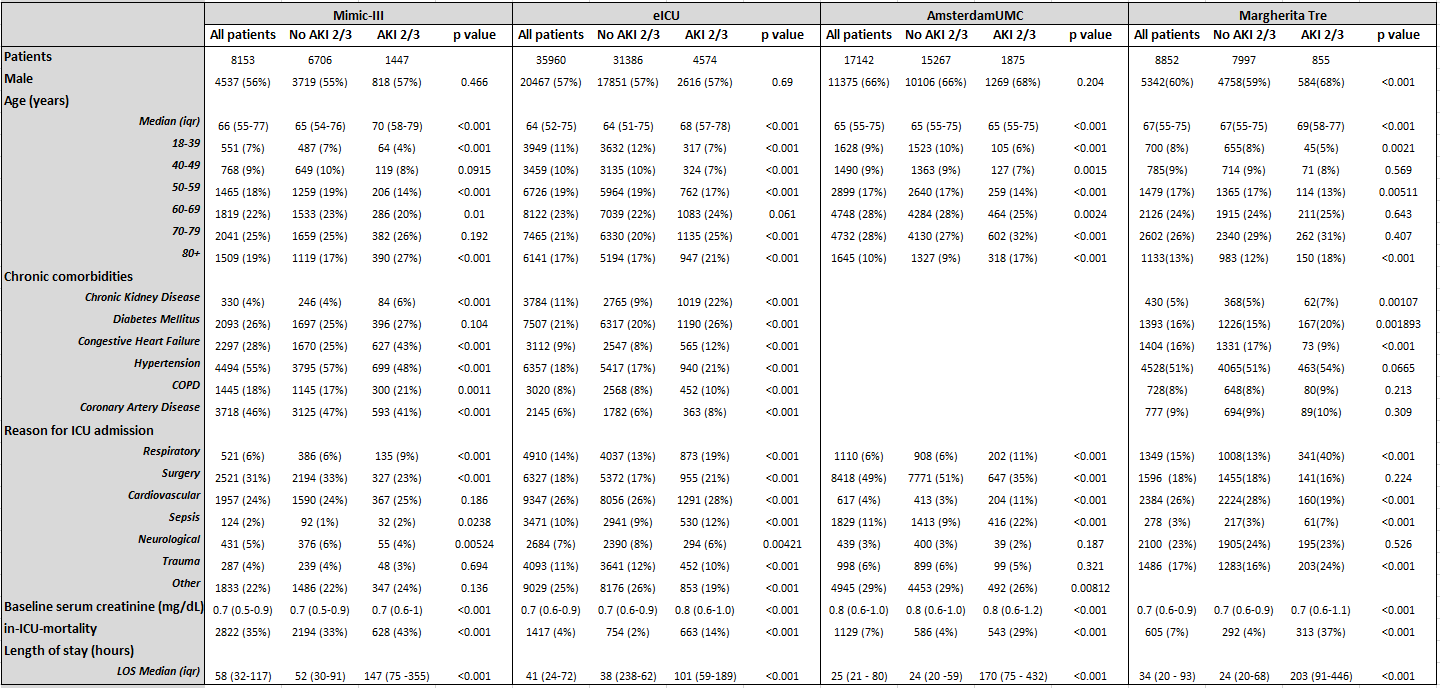


### Acquisition rate of parameters

In the following section, the average acquisition frequency for clinical input parameters employed by our model have been studied. The calculation was performed computing the time-difference among consecutive registered values per parameter per patients and averaged. Subsequently, the median value was extracted **(Table S7, S8).**

The acquisition frequency of clinical parameters in the datasets is comparable with that in the common Intensive Care Unit protocols [2] and can be regarded as representative of real-world scenarios of the target clinical setting, i.e. Intensive Care Unit department.

Differences in the acquisition frequency among datasets might be attributable to the different test executed for the measurements acquisition since both arterial blood test (once every 6-8 hours) and common blood test (once per day) are conducted in ICUs, with no fixed protocols.

Table S7: Median acquisition frequency of clinical variables of each cohort

|  | ***Maria Vittoria Hospital*** | ***Maggiore Hospital*** | ***Juan XXIII Hospital*** |
| --- | --- | --- | --- |
| ***Variable*** | *median acquisition frequency (h) – [iqr]* | *median acquisition frequency (h) – [iqr]* | *median acquisition frequency (h) – [iqr]* |
| Albumin | 145.0 [125.33 - 156.5] | 24.33 [24.0 - 32.0] | 70.45 [42.35 - 123.31] |
| Bun | 24.0 [22.88 - 25.35] | 24.0 [23.24 - 24.0] | 19.21 [16.17 - 21.99] |
| Creatinine | 24.0 [23.0 - 25.13] | 24.0 [23.5 - 24.0] | 20.4 [17.43 - 23.2] |
| Diastolic Pressure | 1.02 [1.01 - 1.04] | 1.02 [1.0 - 1.28] | 1.33 [1.12 - 1.75] |
| Heart rate | 1.02 [1.01 - 1.05] | 1.0 [1.0 - 1.0] | 1.0 [1.0 - 1.01] |
| Hemoglobin | 24.0 [22.5 - 24.48] | 24.0 [23.0 - 24.0] | 15.58 [11.17 - 18.5] |
| Platelets | 24.0 [21.09 - 24.45] | 24.0 [23.0 - 24.0] | 20.4 [16.9 - 23.08] |
| Systolic Pressure | 1.02 [1.0 - 1.04] | 1.02 [1.0 - 1.28] | 1.33 [1.12 - 1.75] |
| Urine Output | 4.47 [3.54 - 5.17] | 3.83 [3.33 - 4.2] | 2.42 [2.28 - 2.88] |
| White Blood Cells | 24.0 [19.12 - 24.45] | 24.0 [23.0 - 24.0] | 20.4 [16.9 - 23.08] |

Table S8: Median acquisition frequency of clinical variables of each cohort

|  | ***AmsterdamUMC*** | | | ***eICU*** | | | ***MargheritaTre*** | | | ***Mimic-III*** | |
| --- | --- | --- | --- | --- | --- | --- | --- | --- | --- | --- | --- |
| ***Variable*** | *median acquisition frequency (h)* | | *IQR* | *median acquisition frequency (h)* | | *IQR* | *median acquisition frequency (h)* | | *IQR* | *median acquisition frequency (h)* | *IQR* |
| Albumin | 22,81 | [15.35, 26.32] | | 23,35 | [13.75, 26.17] | | 24 | [15.92 , 33.6 ] | | 32,29 | [18.92, 74.9] |
| Bun | 19,49 | [12.63, 24.88] | | 18,67 | [11.97, 23.83] | | 22,1 | [15.5 , 24.0 ] | | 16,76 | [12.48, 20.72] |
| Creatinine | 15,92 | [11.83, 19.65] | | 18,61 | [11.94, 23.83] | | 21,5 | [15.27 , 24.04 ] | | 16,72 | [12.44, 20.64] |
| Diastolic Pressure | 0,96 | [0.02, 1.0] | | 0,99 | [0.77, 1.18] | | 1 | [0.97 , 1.93 ] | | 0,74 | [0.61, 0.89] |
| Heart rate | 0,96 | [0.02, 1.0] | | 0,87 | [0.65, 1.0] | | 1 | [0.97 , 1.93 ] | | 0,84 | [0.72, 0.94] |
| Hematocrit | 3,05 | [2.0, 5.5] | | 18,8 | [10.68, 23.92] | | 19,7 | [14.5 , 24.0 ] | | 8,39 | [5.24, 13.42] |
| Hemoglobin | 2,99 | [2.0, 5.41] | | 18,39 | [10.33, 23.9] | | 19,67 | [14.5 , 24.0 ] | | 12,27 | [6.95, 17.98] |
| Platelets | 9,49 | [5.41, 17.65] | | 21,11 | [13.58, 24.03] | | 19,62 | [14.47 , 24.0 ] | | 15,9 | [10.9, 20.5] |
| Systolic Pressure | 0,97 | [0.02, 1.0] | | 0,99 | [0.77, 1.18] | | 1 | [0.97 , 1.93 ] | | 0,75 | [0.63, 0.92] |
| Urine Output | 1,39 | [1.19, 1.68] | | 2,15 | [1.35, 3.4] | | 1,02 | [1.0 , 2.03 ] | | 1,15 | [1.04, 1.38] |
| White Blood Cells | 15,27 | [9.23, 20.55] | | 21,32 | [13.9, 24.04] | | 19,62 | [14.47 , 24.0 ] | | 17,27 | [12.43, 22.21] |

### XGboost vs Random Forest

Performances comparisons among the new AI model and the previous one across retrospective cohorts, are provided in Table S7 with the aim of highlighting differences and benefits. The inclusion of patients with missing data of clinical variables during their ICU stay, resulted in a larger number of patients compared to the previous study [3] The increase in the final ICU stays cohort size is approximately four times (70’107 vs 16’787). The incidence of moderate and severe AKI was not significantly impacted by this cohort size increase, remaining stable except for MargheritaTre datasets where there was an increase from 6% to 10%. On the population derived from the same datasets, the new AI model (based on Xgboost Classifier) performed better in terms of predictive accuracy than previous one (based on Random Forest Classifier): auROC of 0.927 vs 0.884 in the Internal test cohort, 0.939 vs 0.911 in the MargheritaTre external test cohort, 0.911 vs 0.877 in the eICU external test cohort.

Table S9: Results comparison with the ones from the previous study

|  | **XGboost** | | | **RandomForest**[3] | | |
| --- | --- | --- | --- | --- | --- | --- |
| **Dataset** | **n° ICUstays** | **% ICU-A-AKI-2/3** | **auROC**  **(CI)** | **n° ICUstays** | **% ICU-A-AKI (stage 2/3)** | **auROC** |
| Internal Test  [mimicIII, amsterdamUMC] | 5’036 | 13% | 0.929  (0.921,0-938) | 1’749 | 15% | 0.884  (0.864, 0.905) |
| External Test [MargheritaTre] | 8’852 | 10% | 0,939  (0.932,.945) | 1’025 | 6% | 0.911  (0.882, 0.936) |
| External Test  [eICU] | 35’960 | 13% | 0,911  (0.907, 0.915) | 6’985 | 15% | 0.877  (0.868, 0.888) |

### Model performances in different ICU subpopulation of retrospective cohorts

The retrospective nature of the patient population used in the development phase could introduce undesired bias, potentially affecting the model’s fairness. To address this, we conducted additional tests considering age groups, comorbidities, reasons for ICU admission, ICU type, and ethnicity. For each attribute, we computed the Average Odds Difference (AOD) [4], a fairness metric that measures disparities by comparing differences in true positive and false positive rates between groups. An AOD close to 0 indicates fair performance across groups, while values outside the range [-0.1, 0.1] suggest potential bias[5].

Patients from both AmsterdamUMC and Mimic-III databases presented low bias on the attribute age (18-39); Hispanics and patients with Diabetes Mellitus in Mimic-III slightly exceeded the fairness acceptable range (**Fig.S1, Fig.S2**). Concerning the MargheritaTre population, the model showed low bias on CKD patients and those admitted to Neurologic units (**Fig.S3**). eICU Cohort showed a low bias only on the attribute chronic kidney disease (CKD) with a fairness range slightly out of the fairness acceptable range [-0.1,0.1], in addiction to CKDs, also Asian patients presented a fairness value out of the range (**Fig.S4**).


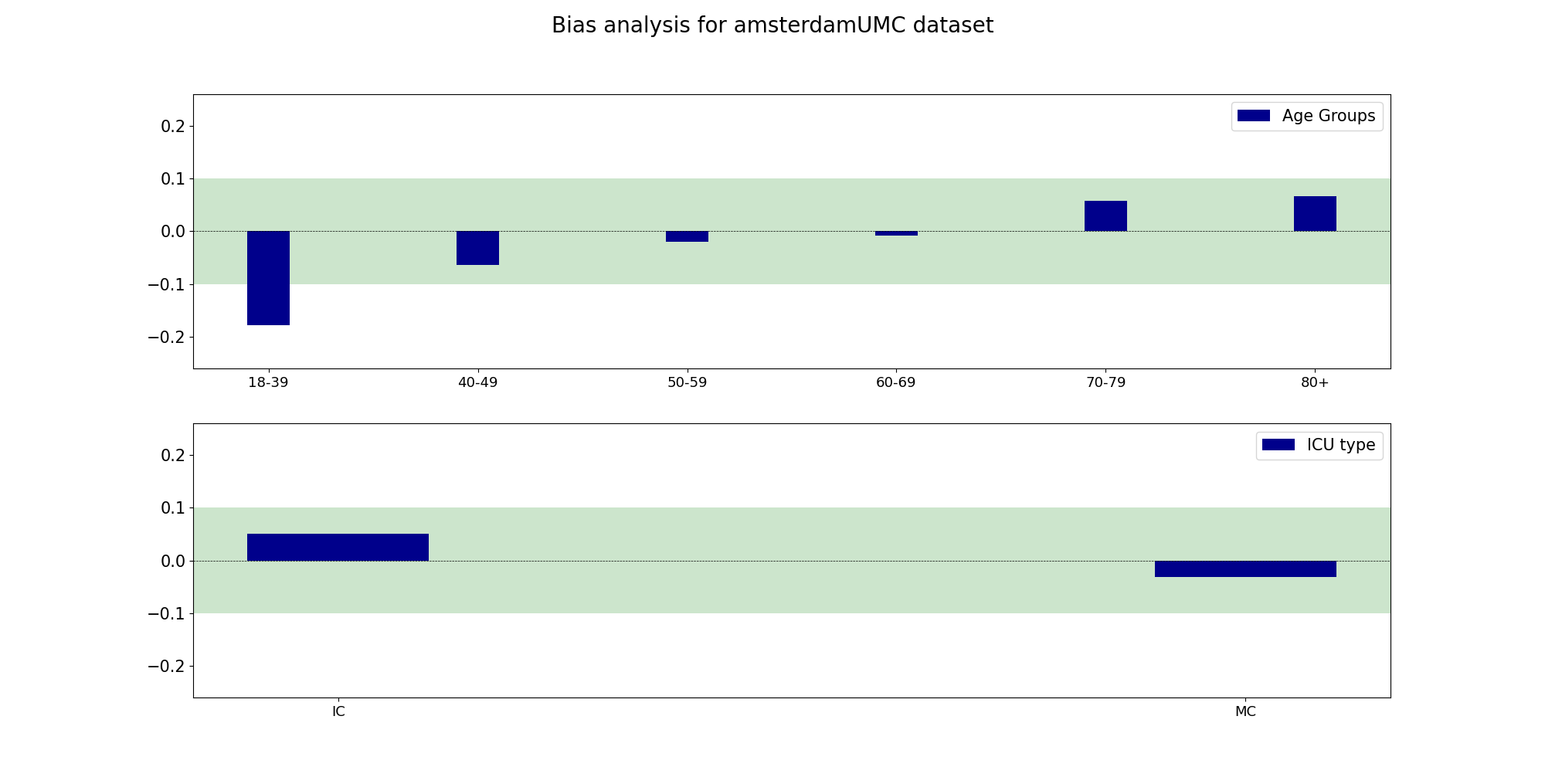


Figure : Bias Detection on AmsterdamUMC population (Internal Test Set)

Legend: fairness range [-0.1,0.1]. Biases are detected when bars exceede the green area.

IC=Intensive Care Unit, MC=Medium Care Unit


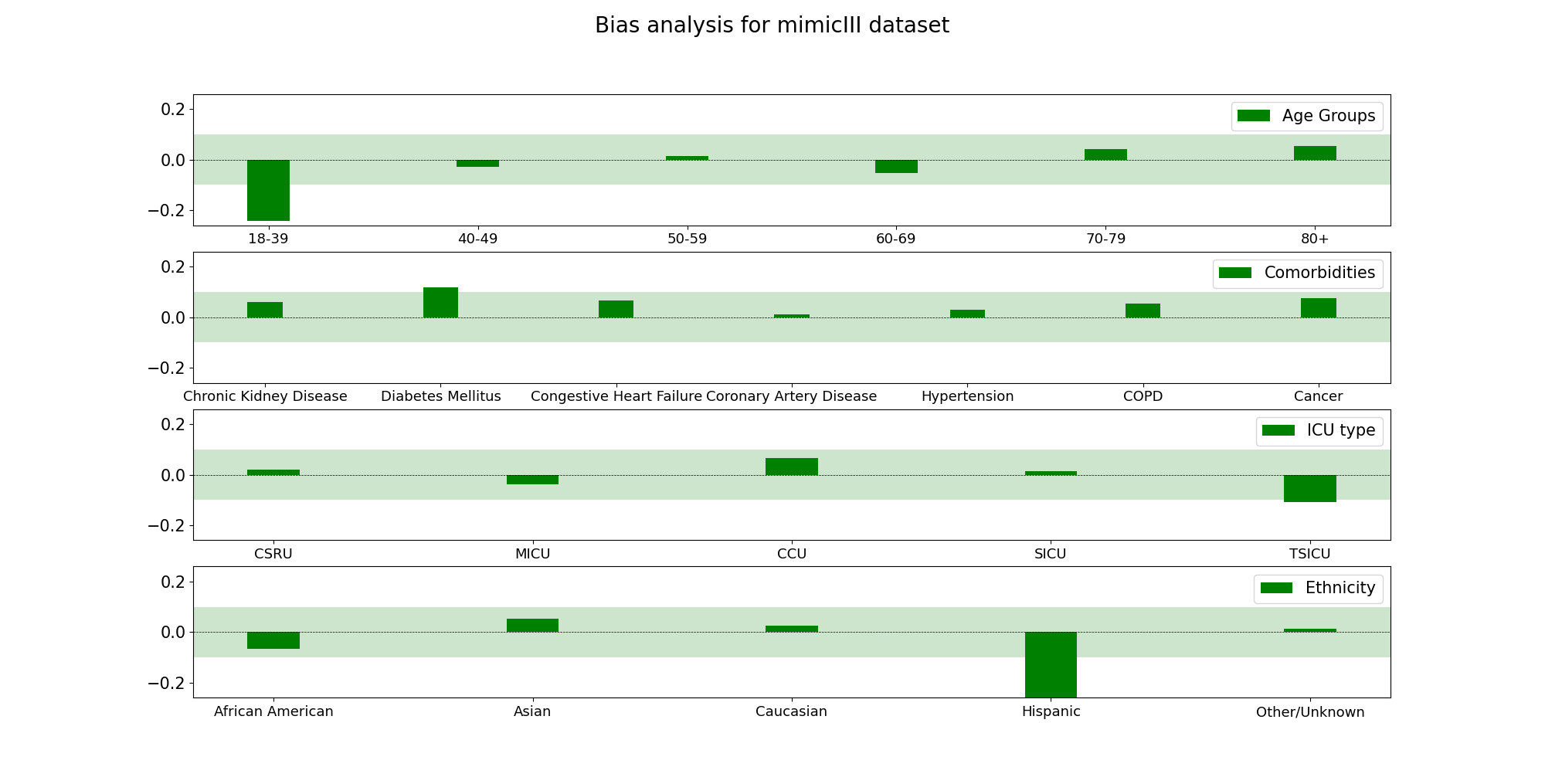


Figure S2: Bias Detection on Mimic-III population (Internal Test Set)

Legend: fairness range [-0.1,0.1]. Biases are detected when bars exceede the green area.

CSRU=Cardio-Surgical Unit, MICU=Medical Unit, SICU=-Surgical Unit, CCU=Coronaryl Unit, TSICU=Trauma-Surgical Unit


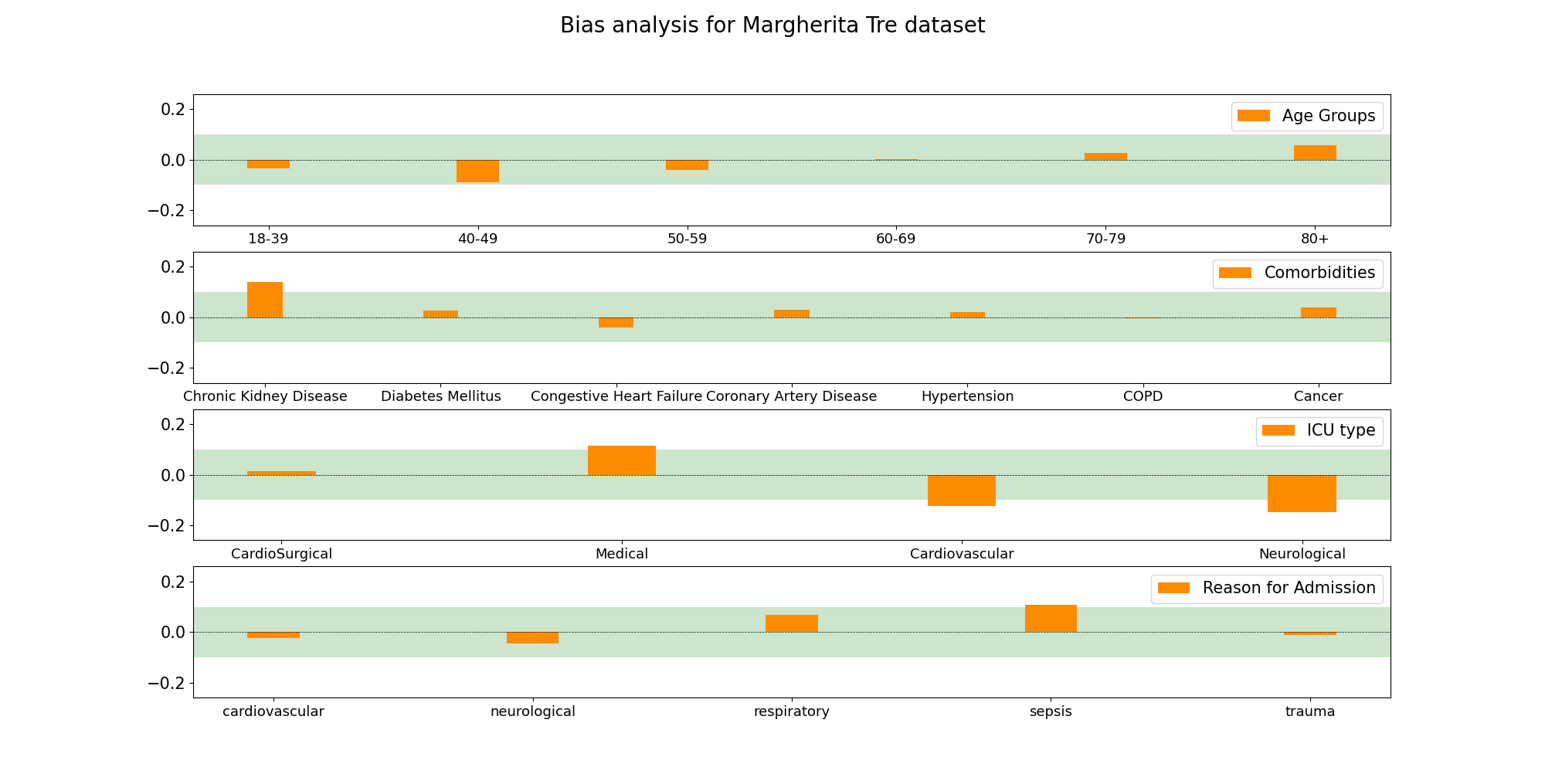


Figure S3: Bias Detection on Margherita Tre population (External Test Set)

Legend: fairness range [-0.1,0.1]. Biases are detected when bars exceede the green area.


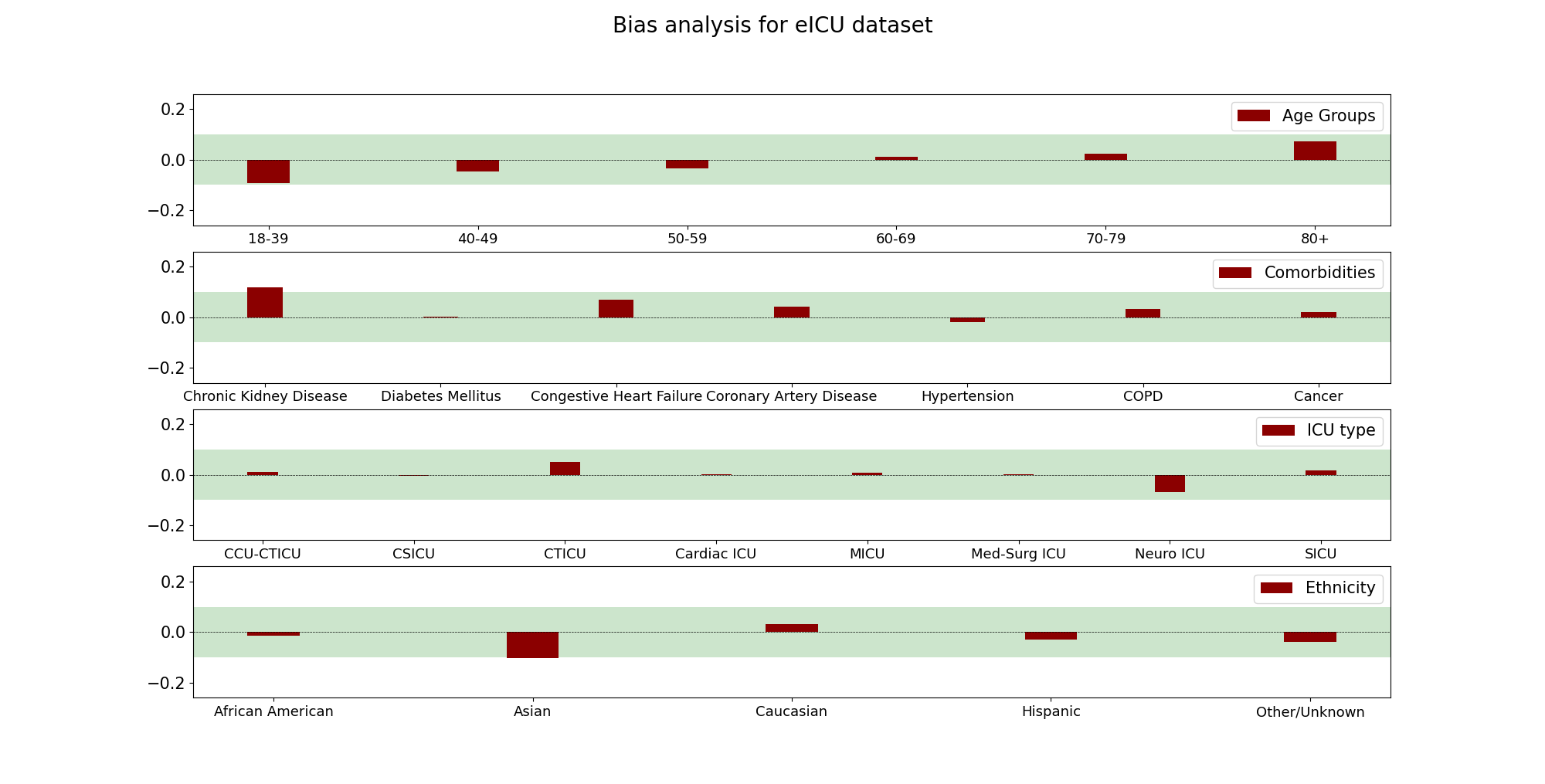


Figure S4: Bias Detection on eICU population (External Test Set)

Legend: fairness range [-0.1,0.1]. Biases are detected when bars exceede the green area.

CSICU=Cardio-Surgical Unit, MICU=Medical Unit, SICU=-Surgical Unit, CCU=Coronaryl Unit, CTICU=Cardio-Toracic Unit, Neuro ICU= Neurological Unit,

### Model’s performances when using different estimates of bsCr

To test the model’s ability in AKI stage 2/3 KDIGO predictions, we conduct a test to evaluate different possible scenarios. It is known that clinicians have multiple ways to detect the basal value of serum creatinine for the identification of AKI when this data is not available. From literature, the use of the creatinine value measured at the *ICU-admission*, the use of a creatinine value derived from Modification of Diet in Renal Disease formula (*MDRD)* formula (eGFR = 75 mL/min/1.73 m2) together with a mixed approach described in the *Opal* study [6] appear to be the most common approaches. All of those have been implemented for retrospective cohorts and the results are shown in **Table S10**.

For the prospective observational study, after the study period concluded, we conducted an additional analysis by reassessing the data of patients and assigning AKI stages according to the KDIGO guidelines, using varying baseline creatinine values. The sensitivity analysis included the following approaches:

- Serum creatinine derived from MDRD formula
- Serum creatinine value at the time of patient enrollment
- Nadir creatinine value measured during the hospitalization

Results are shown in **Table S11.**

Low changes on the auROC metric can be observed over the different datasets, moreover, the averaged PPV remains above 41% in all cohorts at each different bSCr estimation.

This demonstrates that our predictive AI-model maintains its good predictive capabilities with minor fluctuations.

Table S10: Model performances when using different baseline serum creatinine (bSCr) estimation in retrospective cohort

| **Dataset**  **Name** | **Estimation Method** | **bSCr** | **auROC** | **auPR** | **%ICU-A-AKI (stage 2/3)** | **Sensitivity** | **Specificity** | **Lead-Time (mean, h)** | **LR +** | **PPV** |
| --- | --- | --- | --- | --- | --- | --- | --- | --- | --- | --- |
|  | Nadir | 0,77  [0.6, 0.97] | 0,950 | 0,479 | 11,03 | 0,837 | 0,896 | 26 | 8,05 | 0,500 |
| amsterdamUMC | MDRD | 0.975  [0.795-1.0] | 0,940 | 0,500 | 10,068 | 0,804 | 0,908 | 58 | 8,74 | 0,493 |
|  | Opal | 0.938  [0.757-1.153] | 0,950 | 0,480 | 10,441 | 0,848 | 0,892 | 56 | 7,85 | 0,477 |
|  | ICU admission | 0.9  [0.7-1.1] | 0,901 | 0,404 | 15,495 | 0,807 | 0,811 | 76 | 4,27 | 0,439 |
|  | Nadir | 0,72  [0.56, 0.94] | 0,911 | 0,443 | 12,72 | 0,821 | 0,839 | 23 | 5,10 | 0,426 |
| eICU | MDRD | 0.966  [0.785-1.017] | 0,912 | 0,416 | 12,094 | 0,798 | 0,855 | 49 | 5,50 | 0,431 |
|  | Opal | 0.92  [0.72-1.2] | 0,917 | 0,421 | 11,984 | 0,837 | 0,838 | 50 | 5,17 | 0,414 |
|  | ICU admission | 0.89  [0.7-1.16] | 0,918 | 0,421 | 11,876 | 0,840 | 0,838 | 50 | 5,19 | 0,412 |
|  | Nadir | 0,71  [0.58,0.89] | 0,939 | 0. 540 | 9,65 | 0,734 | 0,918 | 10 | 8,95 | 0,492 |
| Margherita Tre | MDRD | 0.968  [0.783- 1.008] | 0,926 | 0.519 | 10,160 | 0.692 | 0.926 | 44 | 9,35 | 0.513 |
|  | Opal | 0.83  [0.69-1.07] | 0.947 | 0.560 | 9.03 | 0.789 | 0.911 | 51 | 8,87 | 0.470 |
|  | ICU admission | 0.83  [0.68-0  108] | 0.947 | 0.554 | 8.96 | 0.785 | 0.911 | 51 | 8,82 | 0.456 |
|  | Nadir | 0,70  [0.5, 0.9] | 0,885 | 0,406 | 17,68 | 0,772 | 0,811 | 36 | 4,08 | 0,467 |
| mimicIII | MDRD | 0.961  [0.778-1.005] | 0,892 | 0,429 | 15,961 | 0,767 | 0,827 | 65 | 4,43 | 0,457 |
|  | Opal | 0.9  [0.7-1.1] | 0,901 | 0,404 | 15,495 | 0,807 | 0,811 | 76 | 4,27 | 0,439 |
|  | ICU admission | 0.8  [0.7-1.1] | 0,895 | 0,380 | 15,131 | 0,798 | 0,810 | 77 | 4,20 | 0,428 |

Table S11: Model performances when using different bSCr estimation for prospective cohort

| **Hospital**  **Name** | **Estimation Method** | **bSCr** | **auROC** | **auPR** | **%ICU-A-AKI (stage 2/3)** | **Sensitivity** | **Specificity** | **Lead-Time (mean, h)** |  | **LR+** | **PPV** |
| --- | --- | --- | --- | --- | --- | --- | --- | --- | --- | --- | --- |
| Maria Vittoria | MDRD | 0.96  [0.77 - 1.0] | 0,960 | 0,437 | 9,62 | 0,968 | 0,700 | 20,43 |  | 3,23 | 0,700 |
|  | ICU admission | 0,981  [0,678-1,27] | 0,865 | 0,448 | 8,65 | 0,947 | 0,555 | 29.00 |  | 2,13 | 0,500 |
|  | Nadir | 0,768  [0,488-0,942] | 0,823 | 0,427 | 21,15 | 0,987 | 0,181 | 18,25 |  | 1,21 | 0,800 |
| Maggiore | MDRD | 0.97  [0.78 - 1.01] | 0,869 | 0,355 | 14,55 | 0,798 | 0,750 | 14,00 |  | 3,19 | 0,387 |
|  | ICU admission | 0,956  [0,65 - 1,148] | 0,889 | 0,435 | 15,45 | 0,806 | 0,824 | 15,21 |  | 4,58 | 0,438 |
|  | Nadir | 0,790  [0,563-0,946] | 0,807 | 0,466 | 19.09 | 0,831 | 0,667 | 15,21 |  | 2,50 | 0,483 |
| Joan XXIII | MDRD | 0.99  [0.8 - 1.04] | 0.820 | 0.428 | 20.00 | 0.761 | 0.826 | 17.63 |  | 4,37 | 0.463 |
|  | ICU admission | 0,796  [0,59-0,93] | 0.822 | 0.326 | 23.47 | 0.784 | 0.704 | 19.58 |  | 2,65 | 0.50 |
|  | Nadir | 0,548  [0,38 -0,68] | 0.646 | 0,218 | 34.78 | 0,80 | 0,425 | 20.82 |  | 1,39 | 0,531 |

### Model Performance in the Prospective Cohort Using Different Eligibility Windows for Data Availability

To assess whether the use of a broader 24-hour data availability window in the prospective cohort introduced selection bias, we performed a sensitivity analysis applying the same 12-hour inclusion criterion used in the retrospective development cohort. This aimed to evaluate the robustness of model performance under a stricter data availability constraint.
We restricted the prospective cohort to patients with both serum creatinine and urine output measurements available within the first 12 hours of ICU admission. We then re-assessed model performance in this subpopulation across all three prospective validation sites, using the same evaluation metrics adopted in the main analysis.

As shown in **Table S12**, using the 12-hour availability window reduced the total number of eligible patients due to exclusion of cases with delayed availability of serum creatinine or urine output measurements. Despite the smaller sample size, the model maintained good discriminatory ability in all centers, with AUROCs above 0.80 supporting the robustness of the model also to this different data availability window.

Table S12: Model Performance in the Prospective Cohort Using Different Eligibility Windows (12 h vs 24 h) for First Creatinine or Urine Output Measurement

| Population Cohorts | **Eligibility Window for First Creatinine/Urine Output (post-ICU admission)** | **n° ICU stays** | **% ICU-A-AKI-2/3** | **auROC (CI)** | **auPR** |
| --- | --- | --- | --- | --- | --- |
| Maria Vittoria Hospital | 12 | 38 | 7.89 | 0.905  (0.809. 1.00) | 0.305 |
|  | 24 | 104 | 9.61 | 0.960  (0.923. 0.990) | 0.448 |
| Maggiore Hospital | 12 | 44 | 18.18 | 0.806  (0.673. 0.962) | 0.396 |
|  | 24 | 110 | 14.55 | 0.869  (0.802. 0.943) | 0.355 |
| Joan XXIII Hospital | 12 | 94 | 20.20 | 0.831  (0.747. 0.929) | 0.404 |
|  | 24 | 115 | 20.00 | 0.820  (0.733. 0.916) | 0.428 |

To ensure transparent reporting of cohort construction, we summarize the patient selection and exclusion process for the prospective cohort. Patients were excluded based on predefined criteria, including missing key variables required for AKI definition (serum creatinine and urine output), missing demographic data, and short ICU length of stay (<24 hours). A detailed breakdown of exclusions is provided in Table S13.

*Table S13. Patient selection and exclusion process Prospective Cohort*

| Population Cohorts | **Initial ICU admissions** | **After exclusion of missing sCr/UO** | **After exclusion of missing demographic data** | **After exclusion of ICU LOS <24h** |
| --- | --- | --- | --- | --- |
| Maria Vittoria Hospital | 260 | 153 | 130 | 104 |
|  |  |  |  |  |
| Maggiore Hospital | 273 | 184 | 144 | 110 |
|  |  |  |  |  |
| Joan XXIII Hospital | 158 | 144 | 142 | 115 |
|  |  |  |  |  |

To complement the description of cohort selection and better characterize the temporal dynamics of AKI development in the prospective cohort, we computed the time from ICU admission to the onset of AKI stage 2/3 KDIGO. The median time and interquartile range (IQR) were calculated for each center in Table S14.

*Table S14: Time from ICU admission to AKI stage 2/3 onset in the prospective cohort*

| Population Cohorts | **Median time to AKI onset (hours)** | **IQR** |
| --- | --- | --- |
| Maria Vittoria Hospital | 41.5 | [37 – 59.5] |
| Maggiore Hospital | 38 | [23.5 – 137] |
| Joan XXIII Hospital | 55 | [23 – 113] |

1. **Timing of Data Availability and First Prediction in Prospective Cohort**

In the prospective cohort, we report the time from ICU admission to the first availability of urine output (UO) and serum creatinine (sCr) across centers. This analysis provides additional context on inter-center variability in data acquisition and its impact on the timing of the first model prediction (Table S15).

Table S15: Timing of data availability for urine output and serum creatinine in the prospective cohort

| **Population Cohorts** | **Mean arrival time of UO (mean ± SD),** | **Mean arrival time of sCr (mean ± SD),** |
| --- | --- | --- |
| **Maria Vittoria Hospital** | 7.9 ± 6.0 | 17.0 ± 6.0 |
| **Maggiore Hospital** | 10.8 ± 10.0 | 11.2 ± 6.0 |
| **Joan XXIII Hospital** | 5.9 ± 5.0 | 4.8 ± 6.0 |

## BIBLIOGRAPHY

[1] E. D. Siew *et al.*, “Commonly used surrogates for baseline renal function affect the classification and prognosis of acute kidney injury,” *Kidney Int*, vol. 77, no. 6, pp. 536–542, Mar. 2010, doi: 10.1038/ki.2009.479.

[2] “MSD MANUAL.”

[3] F. Alfieri *et al.*, “Continuous and early prediction of future moderate and severe Acute Kidney Injury in critically ill patients: Development and multi-centric, multi-national external validation of a machine-learning model,” *PLoS One*, vol. 18, no. 7 July, Jul. 2023, doi: 10.1371/journal.pone.0287398.

[4] “Fairness.”

[5] “AI Fairness 360.”

[6] E. A. J. Hoste *et al.*, “Derivation and validation of cutoffs for clinical use of cell cycle arrest biomarkers,” *Nephrology Dialysis Transplantation*, vol. 29, no. 11, pp. 2054–2061, Nov. 2014, doi: 10.1093/ndt/gfu292.
